# Improved prediction of radiation-induced hypothyroidism in nasopharyngeal carcinoma using pre-treatment CT radiomics

**DOI:** 10.1038/s41598-023-44439-2

**Published:** 2023-10-14

**Authors:** Napat Ritlumlert, Siriporn Wongwattananard, Anussara Prayongrat, Sornjarod Oonsiri, Sarin Kitpanit, Danita Kannarunimit, Chakkapong Chakkabat, Chawalit Lertbutsayanukul, Sira Sriswasdi, Yothin Rakvongthai

**Affiliations:** 1 Biomedical Engineering Program, Faculty of Engineering, Chulalongkorn University, Bangkok, 10330 Thailand; 2 Chulalongkorn University Biomedical Imaging Group, Department of Radiology, Faculty of Medicine, Chulalongkorn University, Bangkok, 10330 Thailand; 3 Division of Radiation Oncology, Department of Radiology, King Chulalongkorn Memorial Hospital, Bangkok, 10330 Thailand; 4 Division of Radiation Oncology, Department of Radiology, Faculty of Medicine, Chulalongkorn University, Bangkok, 10330 Thailand; 5 Center for Artificial Intelligence in Medicine, Research Affairs, Faculty of Medicine, Chulalongkorn University, Bangkok, 10330 Thailand; 6 Center of Excellence in Computational Molecular Biology, Chulalongkorn University, Bangkok, 10330 Thailand; 7 Division of Nuclear Medicine, Department of Radiology, Faculty of Medicine, Chulalongkorn University, Bangkok, 10330 Thailand

**Keywords:** Predictive markers, Cancer, Medical imaging

## Abstract

When planning radiation therapy, late effects due to the treatment should be considered. One of the most common complications of head and neck radiation therapy is hypothyroidism. Although clinical and dosimetric data are routinely used to assess the risk of hypothyroidism after radiation, the outcome is still unsatisfactory. Medical imaging can provide additional information that improves the prediction of hypothyroidism. In this study, pre-treatment computed tomography (CT) radiomics features of the thyroid gland were combined with clinical and dosimetric data from 220 participants to predict the occurrence of hypothyroidism within 2 years after radiation therapy. The findings demonstrated that the addition of CT radiomics consistently and significantly improves upon conventional model, achieving the highest area under the receiver operating characteristic curve (AUCs) of 0.81 ± 0.06 with a random forest model. Hence, pre-treatment thyroid CT imaging provides useful information that have the potential to improve the ability to predict hypothyroidism after nasopharyngeal radiation therapy.

## Introduction

Radiation therapy is a cancer treatment that involves delivering a high dose of radiation to a target volume in the body while minimizing exposure to critical organs to prevent complications. Early and late adverse effects are significant factors when planning radiation therapy due to their impact on the patients' health and daily lives post-treatment. In head and neck malignancies in particularly, the risk and severity of late effects are influenced by the total dose received and the organ exposed^1^. The thyroid gland is highly susceptible to complications because it is radiosensitive and its location anterior to the neck region exposes the organ to radiation beams. This makes hypothyroidism one of the most common side effects of radiation therapy in head and neck tumors that occurs in 15% to 48% of the patients^2,3^.

Currently, laboratory test results and clinical symptoms are used to diagnose radiation-induced hypothyroidism (RIH). Lertbusayanukul et al.^4^ validated a prior report of dose factors in hypothyroidism after intensity-modulated radiation treatment (IMRT) in patients with nasopharyngeal carcinoma (NPC), reporting that a thyroid-stimulating hormone (TSH) level of greater than 1.55 μU/ml and a thyroid volume spared from 60 Gy radiation (VS_60_) of less than 10 cm^3^ were important predictors. Similarly, Peng et al.^5^ showed that the pre-treatment thyroid volume and the percentage of thyroid volume exposed to 30–60 Gy radiation (V_30,60_) can predict RIH with moderate area under the receiver operating characteristic curve (AUCs) of 0.64. Therefore, an effective predictive model is needed to improve treatment planning and reduce the occurrence of RIH.

Medical imaging provides essential information about the pathophysiology of the tumor that is useful for cancer treatment planning, monitoring, and post-therapy evaluation. In addition to qualitative visual inspection of radiological images by clinicians, quantitative numerical data can also be extracted from radiological images via radiomics approaches. Radiomics features describes various metrics of the shape, size, and heterogeneity of the tumor that can complement clinical and dosimetric data when developing predictive machine learning models for clinical applications. Past studies shown the benefits of radiomics information on the prediction of locoregional recurrence, response to treatment, survival, and complications in head and neck cancer patients^6^. Khadija et al.^7^ reported that radiomics features from pre-treatment computed tomography (CT) and magnetic resonance (MR) imagings of salivary glands might be able to reflect the functional states of the glands and are predictive of post-radiation xerostomia. Hence, the incorporation of radiomics features of the thyroid gland should improve the ability to predict RIH compared to conventional methods that rely on only clinical and dosimetric data.

In this study, the occurrence of RIH in nasopharyngeal cancer patients within 2 years after treatment were predicted using radiomics features from pre-treatment contrast-enhanced CT images together with clinical and dosimetric data. Conventional models using only clinical and dosimetric data were compared to the combined models that utilized radiomics features to evaluate the benefits of radiomics features on RIH prediction. Multiple machine learning models were explored to assess the reproducibility of the findings. The results can lead to the development of a pre-treatment planning tool that helps clinicians optimize radiation dosage to manage the risk of RIH.

## Results

Table 1 summarizes the demographic and clinical characteristics of the 220 nasopharyngeal cancer patients, 106 (48.18%) of which developed RIH within 2 years after radiation therapy. The average age was 48.28 ± 11.71 years, with the majority being male (72.27%). Most patients were of clinical staged 3 (51.82%), N stage 2 (51.82%), and T stages 2 or 1 (33.18% and 31.36%, respectively). Two pre-treatment clinical variables, the level of thyroid-stimulating hormone (TSH) and the thyroid volume, were significantly different between the patients with and without RIH (adjusted Mann–Whitney *U* test p-values < 0.05). The average levels of TSH were 2.68 ± 7.08 μU/ml in patients with RIH and 1.83 ± 2.30 μU/ml in patients without RIH. The average volumes of the thyroid glands were 13.23 ± 6.43 cm^3^ in patients with RIH and 15.06 ± 7.07 cm^3^ in patients without RIH. There was no significant difference in dosimetric variables between the two groups of patients as shown in Table 2.

**Table 1:** Patient characteristics.

	Mean ± SD	RIH (N = 106)	No RIH (N = 114)	P-value
Age (years)	48.28 ± 11.71 (18–83)	46.53 ± 11.22 (21–70)	49.90 ± 11.96 (18–83)	0.05
Sex (%)				0.10
Male/female	72.27/27.73	66.98/33.02	77.19/22.81	
T stage (%)				0.36
T1	31.36	33.01	29.82	
T2	33.18	34.91	31.58	
T3	7.73	16.04	19.30	
T4	17.73	16.04	19.30	
N stage (%)				0.06
N0	8.18	4.72	11.40	
N1	28.64	23.58	33.34	
N2	51.82	57.55	46.49	
N3	11.36	14.15	8.77	
Clinical stage (%)				0.73
1	1.82	1.89	1.75	
2	17.27	16.04	18.42	
3	51.82	52.83	50.88	
4a	19.55	17.92	21.05	
4b	9.54	11.32	7.90	
FT4 before treatment (ng/dl)	1.25 ± 0.22 (0.42–1.80)	1.24 ± 0.23 (0.42–1.80)	1.25 ± 0.22 (0.68–1.78)	0.45
TSH before treatment (μU/ml)	2.24 ± 5.19 (0.31–74.34)	2.68 ± 7.08 (0.46–74.34)	1.83 ± 2.30 (0.31–18.99)	< 0.05
Thyroid volume before treatment (cm^3^)	14.78 ± 6.82 (5.00–61.80)	13.23 ± 6.43 (5.50–46.70)	15.06 ± 7.07 (5.00–61.80)	< 0.05
Pituitary volume before treatment (cm^3^)	0.25 ± 0.17 (0.00–1.00)	0.26 ± 0.18 (0.00–1.00)	0.24 ± 0.16 (0.00–0.80)	0.61

**Table 2:** Dosimetric parameters.

Dosimetric parameters	Mean ± SD	RIH (N = 92)	No RIH (N = 103)	P-value
Minimum dose to thyroid gland (Gy)	34.41 ± 7.13 (2.64–7.13)	34.82 ± 6.68 (2.64–53.10)	34.02 ± 7.55 (10.94–51.80)	0.41
Maximum dose to thyroid gland (Gy)	66.34 ± 7.76 (46.69–101.90)	66.93 ± 7.55 (53.60–91.90)	65.79 ± 7.94 (46.69–101.90)	0.19
Mean dose to thyroid gland, TR mean (Gy)	52.36 ± 6.68 (29.80–74.20)	53.05 ± 6.11 (29.80–71.60)	51.72 ± 7.14 (32.80–74.20)	0.10
TR V_40_ (%)	88.93 ± 16.31 (8.90–100.00)	91.16 ± 13.38 (41.20–100.00)	86.86 ± 18.45 (8.90–100.00)	0.19
TR V_50_ (%)	64.93 ± 24.45 (0.00–100.00)	67.08 ± 23.10 (10.00–100.00)	62.94 ± 25.57 (0.00–99.70)	0.24
TR V_60_ (%)	14.89 ± 19.70 (0.00–83.40)	17.34 ± 20.97 (0.00–83.40)	12.62 ± 18.23 (0.00–78.60)	0.08
TR VS_40_ (%)	16.31 ± 21.49 (0.00–71.96)	14.07 ± 20.30 (0.00–56.63)	18.39 ± 22.42 (0.00–71.96)	0.12
TR VS_50_ (%)	44.59 ± 19.44 (0.00–74.20)	46.07 ± 18.64 (0.00–71.60)	43.21 ± 20.13 (0.08–74.20)	0.17
TR VS_60_ (%)	45.83 ± 17.19 (5.49–74.20)	47.42 ± 16.24 (5.49–71.60)	44.35 ± 17.98 (6.73–74.20)	0.12
Minimum dose to pituitary gland (Gy)	45.44 ± 20.17 (4.45–96.70)	44.11 ± 20.51 (4.45–75.00)	46.67 ± 19.86 (9.40–96.70)	0.42
Maximum dose to pituitary gland (Gy)	60.80 ± 15.45 (7.00–102.00)	58.97 ± 16.68 (7.00–84.50)	62.49 ± 14.08 (16.40–102.00)	0.16
Mean dose to pituitary gland (Gy)	52.91 ± 17.68 (0.50–100.60)	51.34 ± 18.14 (6.10–82.50)	54.36 ± 17.19 (0.50–100.60)	0.28
Pit V_50_ (%)	64.98 ± 42.78 (0.00–100.00)	64.33 ± 43.03 (0.00–100.00)	65.58 ± 42.72 (0.00–100.00)	0.85
Pit V_55_ (%)	56.12 ± 44.93 (0.00–100.00)	56.14 ± 45.03 (0.00–100.00)	56.11 ± 45.03 (0.00–100.00)	0.86

TR V_40_, V_45_, V_50_, V_60_ = Percentage of thyroid volume that received at least 40, 45, 50, 60 Gy, respectively.

TR VS_40_, VS_50_, VS_60_ = Percentage of thyroid volume spared from 40, 50, 60 Gy, respectively.

Pit V_50_, V_55_ = Percentage of pituitary volume that received at least 50, 55 Gy, respectively.

A total of 1,288 radiomics features were extracted from pre-treatment contrast-enhanced CT images and categorized into four classes: shape, first-order statistics, texture, and filter-based. The robustness of each radiomics features to the variation in regions of interest drawn by different radiation oncologists were evaluated using intraclass correlation (ICC). Filtering based on ICC values greater than 0.5 and 0.75 reduced the number of radiomics features to 838 and 1026, respectively (Supplementary Table 1). Univariate analysis of radiomics features indicated that while the values of several features significantly differ between patients with and without RIH, they are only moderately predictive of RIH (Supplementary Table 2, AUC = 0.64–0.65). Highly predictive radiomics features include the wavelet-HLL_glcm_MaximumProbability, log-sigma-1-0-mm-3D_ngtdm_Coarseness, wavelet-LLH_ngtdm_Strength, and wavelet-LLH_ngtdm_Strength.

Five machine learning models were trained and cross-validated on different combinations of clinical, dosimetric, and radiomics data (Table 3). The areas under the receiver operating characteristic curve (AUC) of the models that incorporated radiomics data were compared to the performance of the conventional models to assess the benefits of radiomics. In all cases, the addition of radiomics data significantly improved the validation AUCs (adjusted signed rank test p-values < 0.05). The combined logistic regression model which incorporated all data types achieved a validation AUC of 0.80 ± 0.06 compared to the AUCs of up to 0.68 ± 0.07 when only clinical and dosimetric data were used. Similarly, the combined random forest model achieved a validation AUC of 0.81 ± 0.06 compared to the AUCs of up to 0.71 ± 0.06 when only clinical and dosimetric data were used. The highest performing models based on support vector machine (SVM) with radial basis kernel, extreme gradient boosting (XGBoost), and adaptive boosting (AdaBoost) achieved slightly lower validation AUCs of 0.77 ± 0.06, 0.77 ± 0.05, and 0.78 ± 0.05, respectively, but still significantly outperformed the model variants that utilized only clinical and dosimetric data.

**Table 3:** Model performance on the training and validation sets.

Data type			Train AUC	Validation AUC	P-value*
Dose	Clinical	Radiomics			
Logistic regression					
+	−	−	0.66 ± 0.02	0.63 ± 0.06	–
−	+	−	0.67 ± 0.01	0.65 ± 0.07	–
+	+	−	0.74 ± 0.01	0.68 ± 0.07	–
−	−	+	0.82 ± 0.01	0.71 ± 0.07	< 0.05
+	−	+	0.79 ± 0.02	0.71 ± 0.07	< 0.05
−	+	+	0.87 ± 0.01	0.78 ± 0.07	< 0.05
+	+	+	0.88 ± 0.01	0.80 ± 0.06	< 0.05
Random forest					
+	−	−	0.84 ± 0.01	0.51 ± 0.06	–
−	+	−	0.83 ± 0.01	0.69 ± 0.07	–
+	+	−	0.83 ± 0.02	0.71 ± 0.06	–
−	−	+	1.00 ± 0.00	0.78 ± 0.06	< 0.05
+	−	+	1.00 ± 0.00	0.78 ± 0.06	< 0.05
−	+	+	1.00 ± 0.00	0.80 ± 0.06	< 0.05
+	+	+	1.00 ± 0.00	0.81 ± 0.06	< 0.05
Support vector machine					
+	−	−	0.65 ± 0.01	0.62 ± 0.07	–
−	+	−	0.66 ± 0.01	0.59 ± 0.06	–
+	+	−	0.68 ± 0.01	0.63 ± 0.09	–
−	−	+	0.83 ± 0.00	0.70 ± 0.04	< 0.05
+	−	+	0.86 ± 0.01	0.67 ± 0.07	< 0.05
−	+	+	0.85 ± 0.02	0.75 ± 0.08	< 0.05
+	+	+	0.87 ± 0.02	0.77 ± 0.06	< 0.05
XGBoost					
+	−	−	0.64 ± 0.02	0.62 ± 0.07	–
−	+	−	0.77 ± 0.02	0.68 ± 0.07	–
+	+	−	0.72 ± 0.02	0.67 ± 0.07	–
−	−	+	0.98 ± 0.00	0.70 ± 0.06	< 0.05
+	−	+	0.98 ± 0.00	0.71 ± 0.06	< 0.05
−	+	+	0.98 ± 0.00	0.77 ± 0.05	< 0.05
+	+	+	0.99 ± 0.00	0.73 ± 0.07	< 0.05
AdaBoost					
+	−	−	1.00 ± 0.00	0.63 ± 0.08	–
−	+	−	0.76 ± 0.02	0.56 ± 0.09	–
+	+	−	0.87 ± 0.02	0.67 ± 0.08	–
−	−	+	0.98 ± 0.01	0.70 ± 0.07	< 0.05
+	−	+	0.70 ± 0.01	0.70 ± 0.06	< 0.05
−	+	+	1.00 ± 0.00	0.78 ± 0.05	< 0.05
+	+	+	0.93 ± 0.01	0.77 ± 0.04	< 0.05

*Compared with the model utilizing only clinical and dosimetric data.

**Figure 1:**
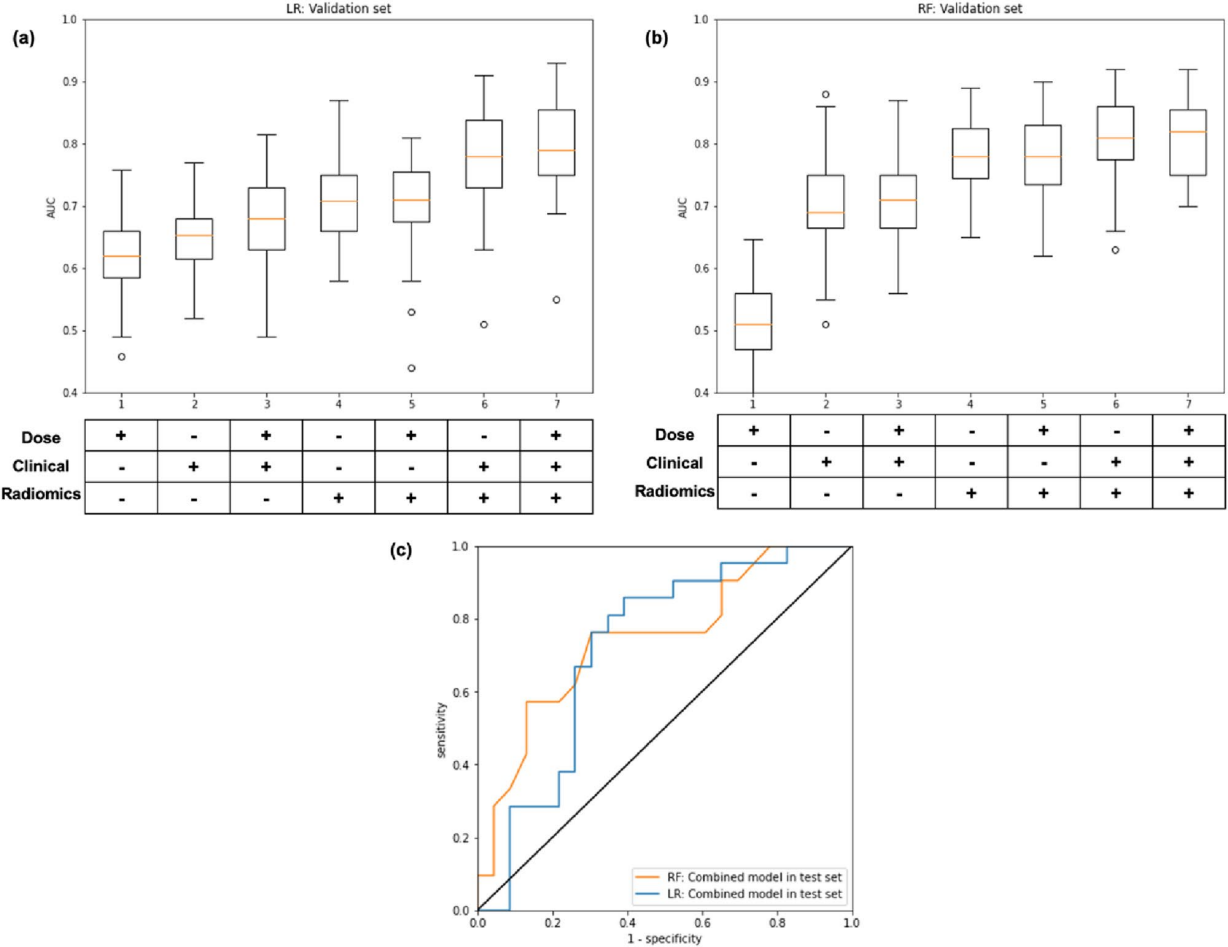
Performances of the logistic regression and random forest models. (**a**) Distributions of the areas under the receiver operating characteristic curve (AUCs) for the logistic regression models trained on different input data combination. (**b**) Similar visualization for the AUCs of the random forest models. (**c**) The receiver operating characteristic curves of the best combined models on the held-out test dataset.

**Figure 2:**
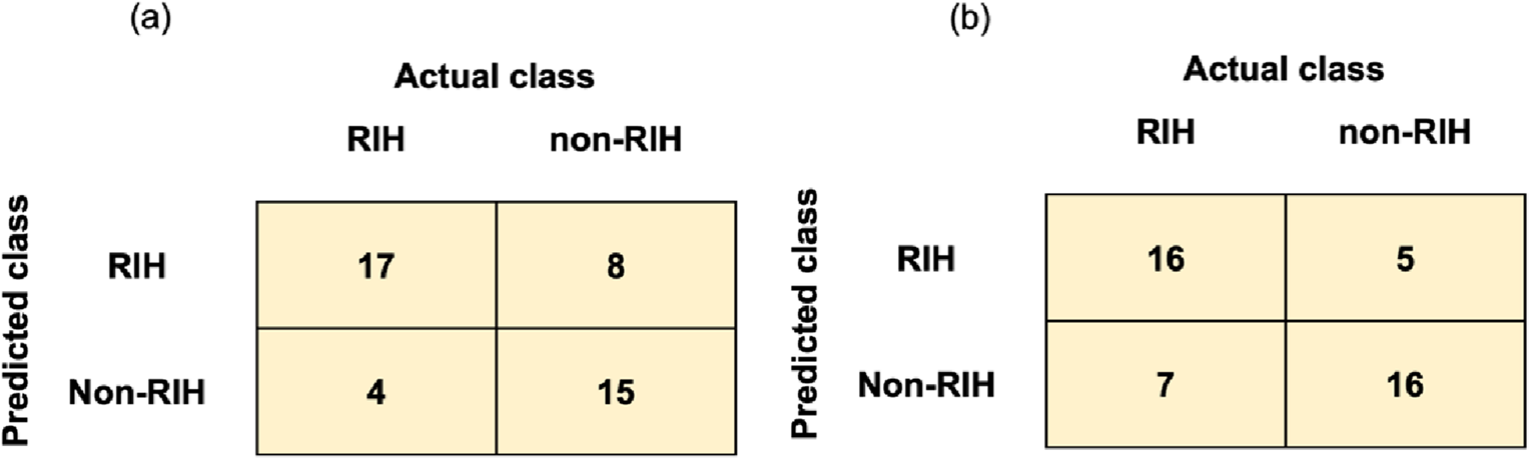
Confusion matrices for the best combined models on the held-out test dataset. (**a**) The combined logistic regression model. (**b**) The combined random forest model.

The top performing models, namely the combined logistic regression model and the combined random forest model, were than evaluated on a held-out test dataset (Fig. 1 and Table 4). The combined models again outperformed the models variants that utilized only clinical and dosimetric data in almost all metrics. The only exception is sensitivity where the combined models did not achieve the best performances. It should be noted that although the combined logistic regression model and the combined random forest model achieved similar AUCs (0.72 and 0.74, respectively), they have different tradeoffs. While the combined random forest model achieved higher sensitivity in the high specificity range (> 0.70), the opposite is true in the intermediate specificity range (Fig. 1c). The confusion matrices also suggested that the combined logistic regression model tends to produce slightly more false positives than false negatives while the combined random forest model behaves in the opposite manner (Fig. 2). Hence, multiple metrics should be considered when selecting the best model and cutoff value.

**Table 4:** Model performance on the held-out test dataset.

Model	AUC	Accuracy	Precision	F1-score	Recall/sensitivity	Specificity
Logistic regression						
Dose	0.49	0.50	0.50	0.50	0.52	0.50
Clinical	0.62	0.64	0.68	0.62	0.86	0.44
Clinical and dose	0.63	0.57	0.57	0.57	0.57	0.57
Radiomic	0.70	0.66	0.70	0.65	0.86	0.48
Radiomic and dose	0.71	0.64	0.64	0.64	0.67	0.61
Radiomic and clinical	0.71	0.70	0.72	0.70	0.81	0.61
Combined	0.72	0.72	0.74	0.73	0.81	0.68
Random forest						
Dose	0.45	0.45	0.45	0.45	0.43	0.50
Clinical	0.62	0.61	0.62	0.61	0.67	0.57
Clinical and dose	0.67	0.68	0.69	0.68	0.76	0.61
Radiomic	0.70	0.61	0.61	0.61	0.57	0.61
Radiomic and dose	0.70	0.64	0.64	0.64	0.67	0.61
Radiomic and clinical	0.73	0.70	0.71	0.70	0.71	0.70
Combined	0.74	0.73	0.73	0.73	0.76	0.70

**Figure 3:**
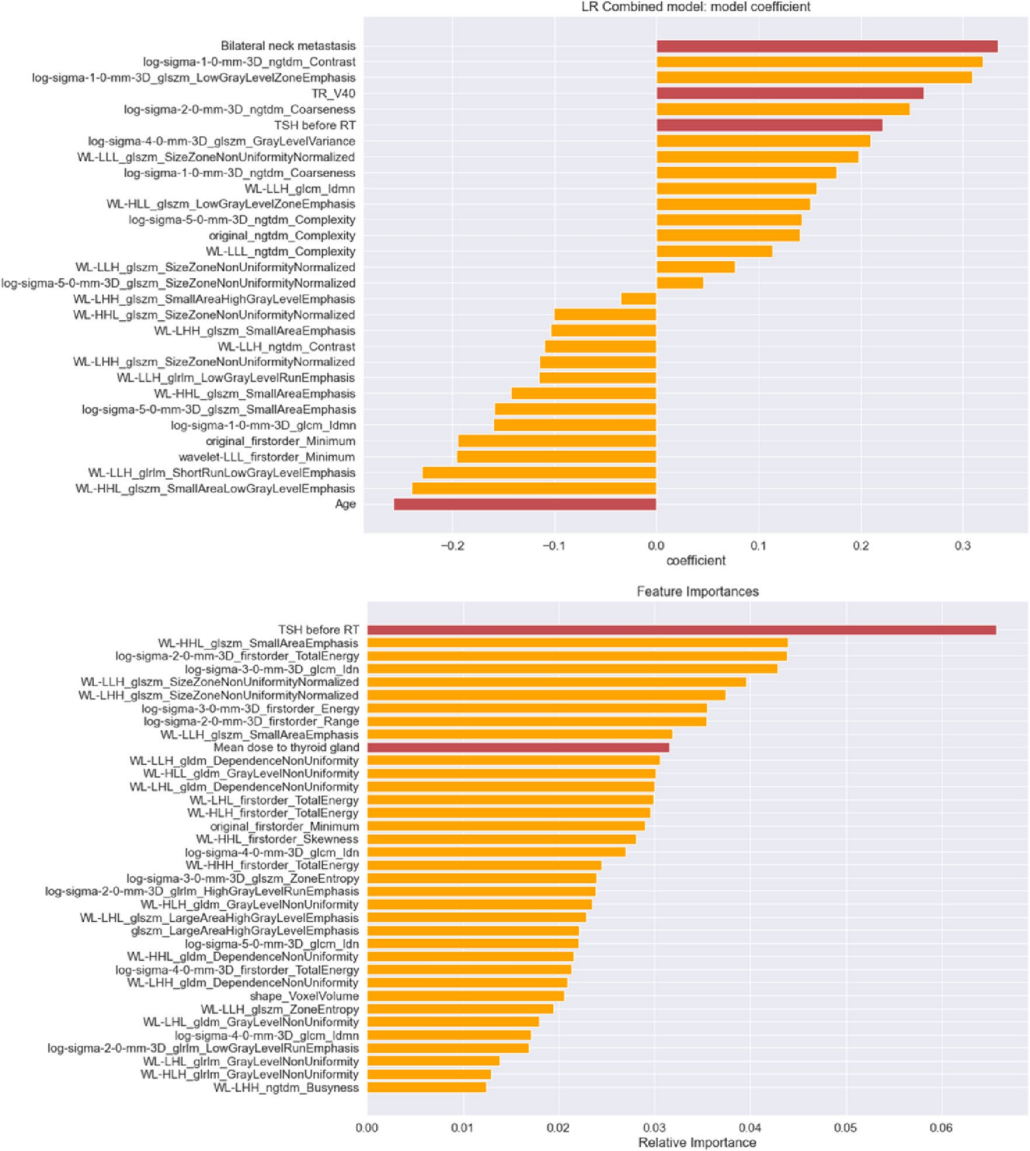
Coefficients and importance of features selected by either the best combined logistic regression model (top) or the best combined random forest model (bottom). Red bars indicate clinical and dosimetric features.

During model development, features that did not strongly contribute to the prediction were iteratively removed. Starting from more than 800 input features, the final combined logistic regression model consisted of three clinical features, namely bilateral neck metastasis status, pre-treatment TSH level, and age, one dosimetric feature, namely the percentage of thyroid volume that received at least 40 (TR V_40_), and 26 radiomics features (Fig. 3). The final combined random forest model contains one clinical feature, namely pre-treatment TSH level, and one dosimetric feature, namely the mean dose to thyroid (TR mean), and 34 radiomics features.

## Discussion

This study aimed to develop a new predictive model for radiation-induced hypothyroidism (RIH) using the combination of clinical, dosimetric, and pre-treatment CT radiomics data and to evaluate the benefits of radiomics data in this context. The results showed that the combined models which incorporated all three data types significantly outperformed the conventional models that utilized only clinical and dosimetric data. In good agreement with prior studies^4,8–10^, the best combined models assigned high importance to the pre-treatment TSH level, age, and metastasis status which have been implicated in RIH. Here, younger age, positive nodes, and high pre-treatment TSH levels were associated with a higher risk of developing RIH. Among dosimetric features, TR V_40_ and TR mean selected by the best combined models had been reported as good predictors for RIH^10,11^. Moreover, the best combined models (validation AUCs of 0.80–0.81 and test AUCs of 0.72–0.74) also compared favorably to recent normal tissue complication probability (NTCP) models^12,13^ which achieved an AUC of only 0.58 on this cohort. These NTCP models considered only age, TR mean, and thyroid volume as predictors.

**Figure 4:**
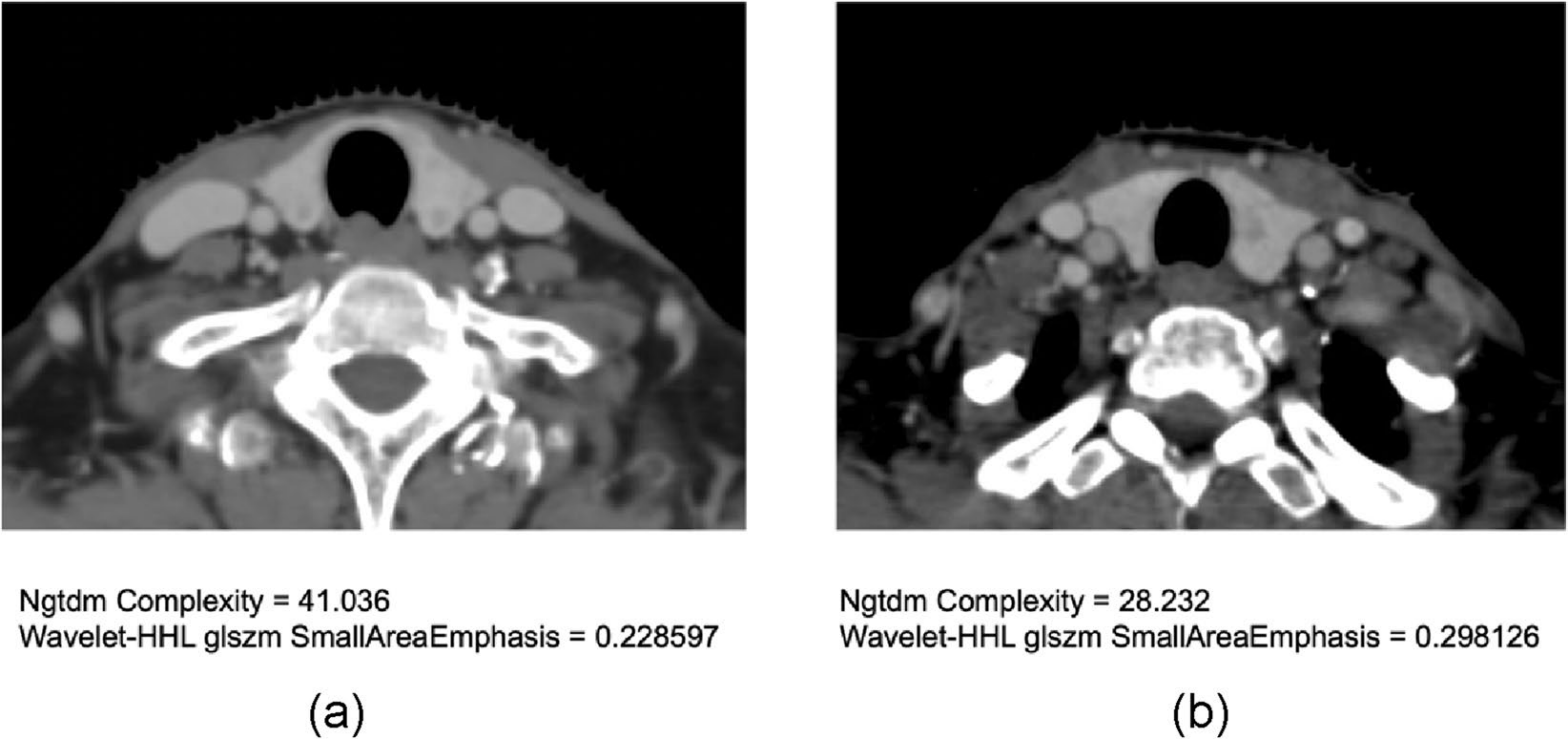
CT images of the thyroid gland with different radiomics feature values. (**a**) Thyroid gland in a patient who developed RIH. (**b**) Thyroid gland in a patient without RIH.

The majority of radiomics features selected by the best combined models came from the texture and filter groups, such as log-sigma_ngtdm_contrast, log-sigma_ngtdm_coarseness, and wavelet-HHL_glszm_SmallAreaEmphasis. Texture features describe the spatial distribution of voxel intensity levels in a region of interest as fine, coarse, grainy, or smooth, which may reflect the pathophysiological status of the organs or malignancies. Ishibashi N et al.^14^ reported that decreased thyroid gland CT intensity might indicate a higher risk of RIH. As subtle changes in the textures of the thyroid may not be discernable by human eyes, radiomics was required to obtain a more precise assessment (Fig. 4).

Although radiomics has been successfully applied to enhance the prediction of radiation-induced complications in various studies^6,7,15^, there are also reports that the incorporation of radiomics features did not significantly improve the prediction of RIH some cancer types, such as oropharyngeal cancer^16^. One possible reason for the contradictory findings could be due to the difference in etiologies, which would influence the association between radiomics features and the clinical status of the tumors. However, our study is not without limitations. First, the retrospective design of the study did not allow for a complete standardization of the CT imaging acquisition protocols. Moreover, RIH requires a long follow-up period which resulted in a relatively small sample size.

In conclusion, while the mechanism of RIH remains unclear, ionizing radiation is expected to damage the thyroid gland, altering the morphology, vessel structure, and immune response of the organ. Our results demonstrated that the combination of information from pre-treatment CT imaging with clinical and dosimetric data significantly improves the prediction of RIH across many performance metrics. We contend that these findings will lead to the development of a better pre-treatment planning tool that helps radiation oncologists optimize dose constraints on the thyroid gland to reduce the risk of hypothyroidism.

## Methods

### Population and sample

This study is retrospective and has been approved by the Ethic Committee at the Faculty of Medicine of Chulalongkorn University (IRB No. 745/61). The need for written informed consent was waived by the same Ethic Committee at the Faculty of Medicine, Chulalongkorn University. All methods were performed in accordance with relevant guidelines and regulations. A total of 220 patients with nasopharyngeal cancer (NPC) whose hypothyroidism statuses were confirmed within 2 years after radiation therapy treatment by the Division of Radiation Oncology, Department of Radiology, King Chulalongkorn Memorial Hospital during the period 2010–2020 were included. The inclusion criteria were age of at least 18 years, treated with definite RT (IMRT or VMAT) with or without chemotherapy, and received a radiation dose of 70 Gy in 33–35 fractions. The patients must also have a normal baseline thyroid function, and no history of pre-existing thyroid disease, thyroid surgery, or radiation therapy in the neck.

### CT image acquisition

All patients underwent a CT simulation before radiation therapy treatment. A 64 detector-row CT simulator was used to acquire CT images (Revolution CT; GE Healthcare, Chicago, IL, USA). Acquisition protocols included a non-contrast phase and a contrast-enhanced phase in helical mode at 120 kV with smart mA by 2.5 mm slice thickness.

### Thyroid segmentation

Manual 3D segmentation of the thyroid gland was performed by radiation oncologists. The region of interest (ROI) covering the thyroid gland in contrast-enhanced CT images were drawn using the Eclipse Contouring software (Varian Medical System, Inc: version 15.5). To assess the robustness of each radiomics feature, an intraclass correlation (ICC) test was performed by having three radiologists segment the same set of thirty randomly selected patients. Robust radiomics features should maintain the same values across ROIs delineated by different radiologists for the same CT image. ICC cutoffs of 0.5 and 0.75 were then applied to select robust radiomics features.

### Radiomics features extraction

Radiomics features were extracted from contrast-enhanced CT images using the PyRadiomics package (version 3.0) through the 3D slicer software (version 4.11.2)^17,18^. There were 14 shape-based features, 18 first-order statistics features, 73 texture-based features, and 1183 filter-based features. The bin width parameter was varied between 0.05, 0.1, 0.15, and 0.2.

### Clinical and dosimetric data collection

Clinical variables were collected from the hospital information system of the King Chulalongkorn Memorial Hospital. Dosimetric variables were calculated from the dose volume histogram, namely V_40_, V_50_, V_60_, Pit_50_, Pit_55_ (V_x_: Percentage of thyroid volume that has received at least × Gy radiation, Pit_x_: Percentage of pituitary volume that has received at least × Gy radiation), VS_40_, VS_50_, VS_60_ (VS_x_: Percentage of thyroid volume preserved from × Gy of radiation), the mean dose of thyroid and pituitary gland, the maximum dose of thyroid and pituitary gland, and the minimum dose of thyroid and pituitary gland dose, via the treatment planning system.

### Model development

**Figure 5:**
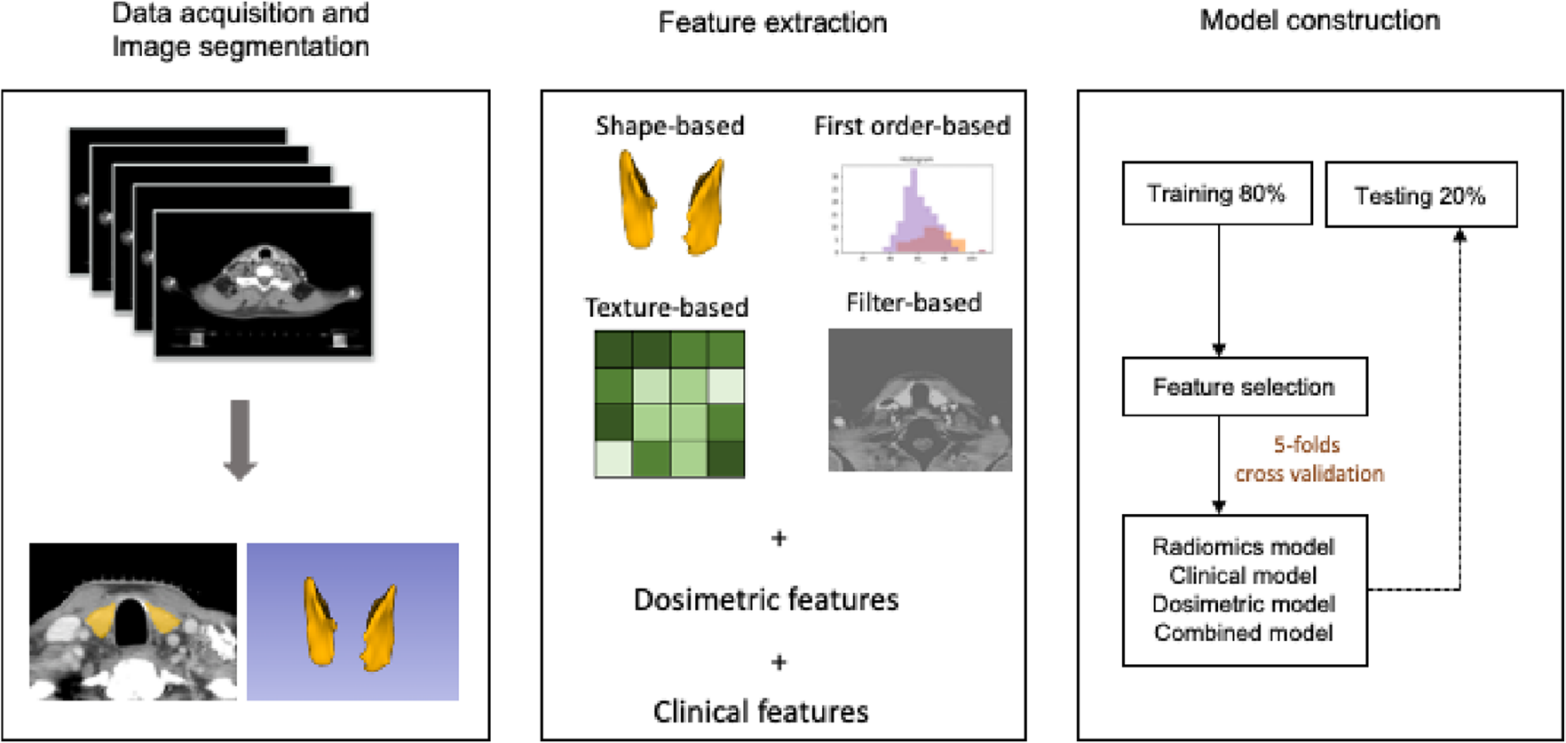
Data acquisition and model development workflow.

The dataset was first divided into training (80%) and testing (20%). Then during model development, the training set was further divided into 5 equal partitions to perform a fivefold cross-validation. The fivefold cross-validation process was repeated 20 times with different random partitioning of the training set. Five machine learning model families, namely regularized logistic regression, random forest, support vector machine (SVM), gradient boosting trees (XGBoost), and adaptive boosting (AdaBoost) were developed through the scikit-learn and xgboost packages^19,20^. Multiple combinations of clinical data, dosimetric data, and radiomics data were considered as inputs. For each initial set of input features, recursive feature elimination was performed to iteratively remove unimportant features that do not strongly contribute to the prediction. The area under the receiver operating characteristic curve (AUC) was used to rank the performance of the models. Figure 5 summarized data acquisition and model development workflow.

### Statistical analysis

The mean and standard deviation (SD) values were calculated for continuous variables, while the counts and percentages were used to summarize categorical features. The differences in feature values between patients with and without RIH were evaluated using the Mann–Whitney *U* tests and Chi-square tests. The difference in predictive performance of the models were evaluated using signed rank tests. The Benjamini–Hochberg procedure was performed to control for multiple testing. A *p*-value cutoff of 0.05 was set to define statistical significance.

### Supplementary Information

Supplementary Tables.

## Data Availability

The data and code sufficient to produce the results in this study are available in [Radiation-induced hypothyroidism] at https://github.com/Amisnapat/Radiation-induced-hypothyroidism/tree/main/code%20RIH. Other raw data are available from the corresponding author (Yothin Rakvongthai, yothin.r@chula.ac.th) upon request.
