# Supplementary material for: Improved prediction of radiation-induced hypothyroidism in nasopharyngeal carcinoma using pre-treatment CT radiomics
Source: Sci Rep. 2023 Oct 14;13:17437. doi: 10.1038/s41598-023-44439-2 (PMC10576799; doi:10.1038/s41598-023-44439-2)
Supplement: Supplementary file 1 — Supplementary Tables. [file 41598_2023_44439_MOESM1_ESM.docx]

**Supplementary Information**

**Table S1 Radiomics features from intra-class correlation**

| **Feature class** | **No. of features** | **ICC > 0.75** | **ICC > 0.50** |
| --- | --- | --- | --- |
| Shape based | 14 | 4 | 5 |
| First order statistic | 18 | 14 | 17 |
| Texture based | 73 | 44 | 63 |
| Filtered-based | 1,183 | 776 | 941 |
| Total | 1288 | 838 | 1026 |

#### Table S2 Radiomics feature univariate analysis

| **Radiomics features** | **mean±SD** | **RIH** | **non-RIH** | **p-value** | **AUC** |
| --- | --- | --- | --- | --- | --- |
| BW 0.05: wavelet-HLL_glcm_MaximumProbability | 42.23 x 10^-2^ ± 0.03 | 43.23 x 10^-2^ ± 0.04 | 41.31 x 10^-2^ ± 0.03 | < 0.05 | 0.64 |
| BW 0.1:  log-sigma-1-0-mm-3D_ngtdm_Coarseness | 13.99 x 10^-5^ ± 0.00 | 15.39 x 10^-5^ ± 0.00 | 12.68 x 10^-5^ ± 0.00 | < 0.05 | 0.64 |
| BW 0.15:  wavelet-LLH_ngtdm_Strength | 9.22 x 10^-5^ ± 0.00 | 10.30 x 10^-5^ ± 0.00 | 8.18 x 10^-5^ ± 0.00 | < 0.05 | 0.65 |
| BW 0.2:  wavelet-LLH_ngtdm_Strength | 8.91 x 10^-5^ ± 0.00 | 9.88 x 10^-5^ ± 0.00 | 8.02 x 10^-5^ ± 0.00 | < 0.05 | 0.65 |
